# Switching from Flash Glucose Monitoring to Continuous Glucose Monitoring on Hypoglycemia in Adults with Type 1 Diabetes at High Hypoglycemia Risk: The Extension Phase of the I HART CGM Study

**DOI:** 10.1089/dia.2018.0252

**Published:** 2018-10-25

**Authors:** Monika Reddy, Narvada Jugnee, Sinthuka Anantharaja, Nick Oliver

**Affiliations:** Division of Diabetes, Endocrinology and Metabolism, Imperial College, London, United Kingdom.

**Keywords:** Continuous Glucose monitoring, Flash glucose monitoring, Type 1 diabetes, Hypoglycemia.

## Abstract

***Background:*** The I HART CGM study showed that real-time continuous glucose monitoring (RT-CGM) has greater beneficial impact on hypoglycemia than intermittent flash glucose monitoring (flash) in adults with type 1 diabetes (T1D) at high risk. The impact of continuing RT-CGM or switching from flash to RT-CGM for another 8 weeks was then evaluated.

***Methods:*** Prospective randomized parallel group study with an extension phase. After a 2-week run-in with blinded CGM, participants were randomized to either RT-CGM or flash for 8 weeks. All participants were then given the option to continue with RT-CGM for another 8 weeks. Glycemic outcomes at 8 weeks are compared with the 16-week endpoint.

***Results:*** Forty adults with T1D on intensified multiple daily insulin injections and with impaired awareness of hypoglycemia or a recent episode of severe hypoglycemia were included (40% female, median [IQR] age 49.5 [37.5–63.5] years, diabetes duration 30.0 [21.0–36.5] years, HbA1c 56 [48–63] mmol/mol, and Gold Score 5 [4–5]), of whom 36 completed the final 16-week extension. There was a significant reduction in percentage time in hypoglycemia (<3.0 mmol/L) in the group switching from flash to RT-CGM (from 5.0 [3.7–8.6]% to 0.8 [0.4–1.9]%, *P* = 0.0001), whereas no change was observed in the RT-CGM group continuing with the additional 8 weeks of RT-CGM (1.3 [0.4–2.8] vs. 1.3 [0.8–2.5], *P* = 0.82). Time in target (3.9–10 mmol/L) increased in the flash group after switching to RT-CGM (60.0 [54.5–67.8] vs. 67.4 [56.3–72.4], *P* = 0.02) and remained the same in the RT-CGM group that continued with RT-CGM (65.9 [54.1–74.8] vs. 64.9 [49.2–73.9], *P* = 0.64).

***Conclusions:*** Our data suggest that switching from flash to RT-CGM has a significant beneficial impact on hypoglycemia outcomes and that continued use of RT-CGM maintains hypoglycemia risk benefit in this high-risk population.

## Introduction

Hypoglycemia is a serious acute metabolic complication of type 1 diabetes (T1D).^1^ Recurrent hypoglycemia may lead to impaired awareness of hypoglycemia (inability to recognize symptoms of hypoglycemia) and severe hypoglycemia (requiring third-party assistance to treat), both of which are associated with increased morbidity and mortality.^2–5^ Hypoglycemia, especially severe hypoglycemia, adds a significant burden to the health cost worldwide.^6^

Capillary finger-prick blood glucose testing remains the mainstay of self-monitoring of blood glucose (SMBG) among people with T1D, but uptake of intermittent flash glucose monitoring (flash) and real-time continuous glucose monitoring (RT-CGM) devices is increasing.

RT-CGM has been shown to improve HbA1c and reduce hypoglycemia in people with T1D established on pump therapy and multiple daily injections (MDI) of insulin with suboptimal glycemic control.^7–11^ RT-CGM also increases time spent in normoglycemia and reduces severe hypoglycemia in T1D participants with impaired awareness of hypoglycemia, compared with SMBG,^12,13^ and reduces diabetes-related distress.^14^ The largest randomized controlled trial (RCT) evaluating flash versus standard care is the IMPACT study that included T1D participants with HbA1c close to target and intact hypoglycemia awareness on MDI of insulin or pump therapy. Flash use was associated with a 38% reduction in time spent in hypoglycemia (<3.9 mmol/L [70 mg/dL]) and HbA1c remained unchanged after 6 months.^15^

Both RT-CGM and flash glucose monitoring devices measure interstitial fluid glucose, but only RT-CGM has alarms to alert users to the potential risk of impending hypo- or hyperglycemia. With flash, a real-time glucose value and 8 h of retrospective data with a trend line can be viewed after physically scanning the sensor. Of the current commercially available interstitial glucose monitoring devices, only the Freestyle Libre Flash Glucose Monitoring System and Dexcom G5 and G6 RT-CGM are licensed to be used nonadjunctively (not requiring confirmation with capillary blood glucose) for insulin treatment decisions. In the United Kingdom, NHS England has endorsed guidance for flash, written by the Regional Medicines Optimisation Committee (RMOC),^16^ that supports the use of flash in people with T1D who undertake intensive monitoring (>8 times) daily; those who fit the current NICE criteria for insulin pump therapy (HbA1c >8.5%) or disabling hypoglycemia (NICE TA151); those who have recently developed impaired awareness of hypoglycemia; if frequent (>2/year) admission with diabetic ketoacidosis or hypoglycemia; and those who require third-party assistance to carry out recommended monitoring. These indications overlap with the NICE guidelines supporting the use of CGM in adults with T1D, which are as follows: >1 episode of severe hypoglycemia/year; complete loss of awareness of hypoglycemia; frequent (>2 episodes/week) asymptomatic hypoglycemia that is causing problems with daily activities; extreme fear of hypoglycemia; and HbA1c ≥9% despite testing ≥10 times/day.^17^

The I HART CGM study was the first head-to-head RCT comparing flash (Freestyle Libre) to RT-CGM (Dexcom G5) in people with T1D at high risk of hypoglycemia and showed a significantly greater reduction in percentage of time spent in hypoglycemia in the RT-CGM group compared with the flash group over 8 weeks; however, no significant differences between the groups in Gold Score or HbA_1c_ from baseline to endpoint were observed.^18^ All participants were given the opportunity to participate in an 8-week extension phase evaluating the impact of switching from flash to RT-CGM and continuing with RT-CGM in those already on RT-CGM to establish its impact on extended use. In this article, we present the glycemic outcomes from the I HART CGM study extension phase, and throughout the article, the study groups are referred to as the flash group and RT-CGM group according to randomization at baseline.

## Methods

### Participants and study design

The I HART CGM study is a randomized, nonmasked parallel group study with an extension phase at a single diabetes specialist site in the United Kingdom. Ethics approval was obtained from the London - Hampstead Research Ethics Committee (Reference no.: 15/LO/1679). The inclusion criteria were as follows: age ≥18 years, T1D (confirmed by a fasting c-peptide <200 pmol/L and clinical features) ≥3 years, on an intensified MDI regimen for >6 months, and a severe hypoglycemic event in the last 12 months requiring third-party assistance or a Gold Score ≥4. All participants had undergone T1D education, either as a group or in a one to one session with a specialist educator. Participants were excluded if they had used CGM or flash within the last six months (except short periods of diagnostic blinded use under clinic supervision), used regular paracetamol, were pregnant or planning pregnancy, breastfeeding, enrolled in other clinical trials, had active malignancy or were under investigation for malignancy, had severe visual impairment, or reduced manual dexterity. Written informed consent was obtained from all participants.

### Procedures

**FIG. 1.**
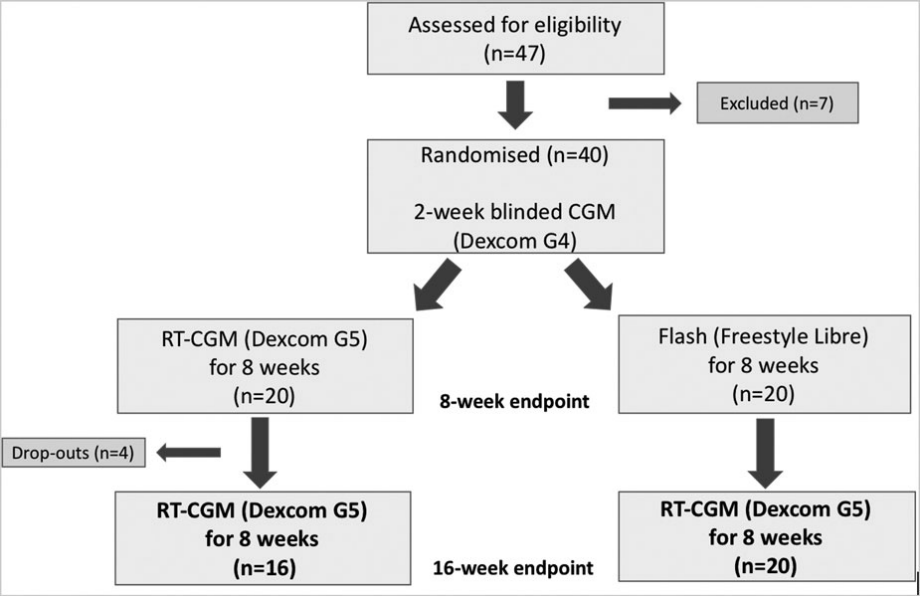
Study design and participant recruitment.

Screening included a medical history, a physical examination, electrocardiogram, fasting venous blood tests (HbA1c, plasma glucose, urea and electrolytes, cortisol, and serum C-peptide), urine for urine microalbuminuria and pregnancy test in women of childbearing age, and validated questionnaires (Gold Score, Hypoglycemia Fear Score II [HFS-II], and Problem Areas in Diabetes [PAID] questionnaires). A brief T1D education refresher was provided. Participants then commenced a 2-week run-in phase using the Dexcom G4 sensor with a blinded receiver running the advanced “505” algorithm that stores glucose data but does not make them available to the participant. The sensor was calibrated to capillary blood glucose values twice daily as per the manufacturer's guidance. Participants were randomly assigned to CGM (Dexcom G5) or flash glucose monitoring (Abbott Freestyle Libre) in a 1:1 ratio by an online randomization tool (www.sealedenvelope.com). Randomization was stratified by HbA1c (HbA1c <58 or ≥58 mmol/mol). Participants then received standardized CGM education for the RT-CGM or flash devices and both devices were used in accordance with product licenses. Participants were instructed to test their capillary blood glucose if they experienced symptoms of hypo- or hyperglycemia, in case of temporary sensor signal loss or sensor failure. The initial intervention period was 8 weeks and participants were given an option at the end of 8 weeks to continue with RT-CGM (Dexcom G5) for another 8 weeks, irrespective of which device they were randomized to for the initial 8 weeks of the study. The complete study design is illustrated in Figure 1 and this article focuses on the extension phase of the study (weeks 9–16). The details of study visits for the first 8-week intervention period have been published.^18^ Those participants who decided to participate in the 8-week extension phase using RT-CGM (Dexcom G5), using either the G5 receiver or the G5 app on their smartphone, entered this phase immediately after completing the first 8-week intervention and those initially randomized to flash were given standardized RT-CGM education. Participants then attended the clinical research facility after 8 weeks (at 16 weeks from initial study start) for a venous blood test for HbA1c and completed the Gold Score, HFS-II, and PAID questionnaires. CGM data were downloaded from their G5 receiver to Diasend (for app users, data upload was automatic). The participants were provided with a contact number for technical support, but insulin titration decisions were made by the participant throughout the study.

### Statistical analysis

Outcomes for the I HART CGM extension phase were percentage time spent in hypoglycemia (<2.8, <3.0, and 3.9 mmol/L), percentage time in euglycemia (3.9–7.8 mmol/L), percentage time spent in target (3.9–10 mmol/L), percentage time spent in hyperglycemia >7.8, >10, and >15 mmol/L, severe hypoglycemia (requiring third-party assistance to treat), hypoglycemia risk, HbA1c, and Gold Score. Data were analyzed using Stata v14 (StataCorp, TX). The majority of variables were non-normally distributed and summary statistics therefore presented as median (IQR). For the extension phase, the change from the 8-week endpoint outcomes of the randomized phase to the 16-week endpoint of the extension phase was calculated for each group and the difference in change between groups is analyzed.

All outcomes from the extension phase were considered to be secondary outcomes and comparisons were considered hypothesis generating and informative. The Wilcoxon matched-pair signed rank test was used for analysis of changes within each group and the Wilcoxon rank sum test used for comparing changes between groups. Analysis was by intention to treat. The 8-week endpoint glucose data were taken from the last 28 days of the initial treatment period and 16-week endpoint outcomes calculated from the last 28 days of the final treatment period. All outcomes are based on comparisons of the 8-week endpoint and 16-week endpoint unless mentioned otherwise. The study is registered at ClinicalTrials.gov, number NCT03028220.

## Results

Thirty-six adults with T1D (all participants had a c-peptide of less than 200 pmol/L) on intensified multiple daily insulin injections and with impaired awareness of hypoglycemia or a recent episode of severe hypoglycemia were included. Of the initial 40 participants included in the I HART CGM study, four participants in the RT-CGM group did not complete the second 8-week treatment period with RT-CGM (one participant decided not to partake in the second treatment period (no reason given), one participant lost the transmitter during the treatment period and did not inform the study team, one participant could not commit to the study due to work, and one participant did not comply with the study protocol and was excluded from the study). An overview of the recruitment is given in Figure 1. For outcomes derived from CGM data, *n* = 15 were analyzed in the RT-CGM group due to loss of the initial 8-week intervention CGM data for one participant (uploading error).

There was a significant reduction in percentage time in clinically relevant hypoglycemia (<3.0 mmol/L) in the flash group after switching to RT-CGM (5.0 [3.7–8.6] vs. 0.8 [0.4–1.9], *P* < 0.001) and no change was observed in the RT-CGM group that continued with RT-CGM (1.3 [0.4–2.8] vs. 1.3 [0.8–2.5], *P* = 0.82). A significant difference in reduction between the groups was observed (*P* < 0.001). The median difference, from the 8-week endpoint to the 16-week endpoint, between groups was significant for all predefined thresholds of hypoglycemia when switching from flash to RT-CGM.

**FIG. 2.**
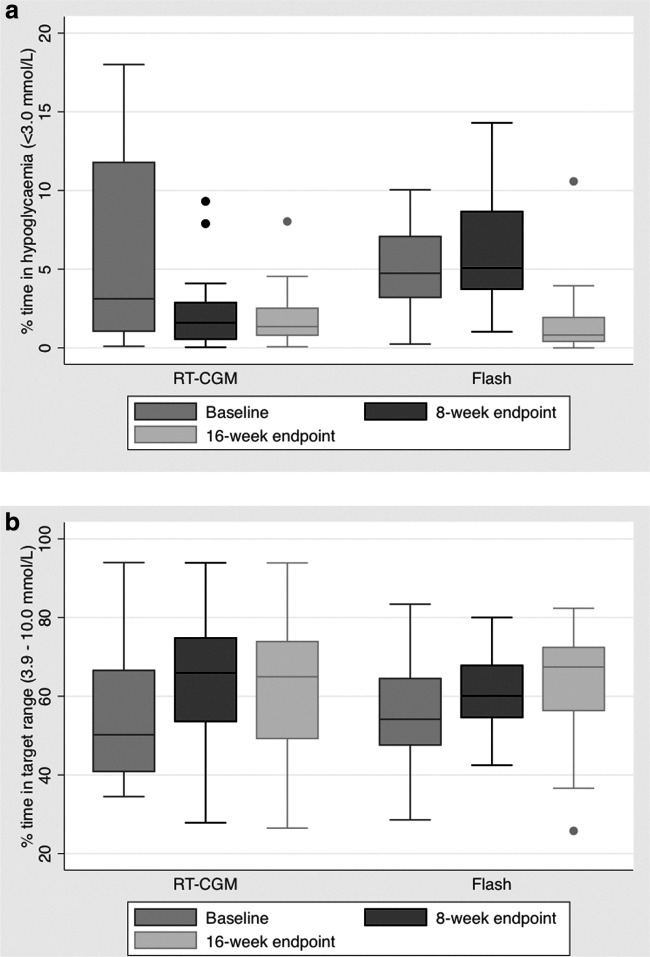
Percentage time in hypoglycemia (<3.0 mmol/L) **(a)** and in target range (3.9–10 mmol/L) **(b)** at baseline (weeks −2 to 0), at 8-week endpoint (weeks 4–8), and at 16-week endpoint. The bar charts represent median (IQR) and the range (minimum to maximum).

There was no significant change in HbA1c within or between groups. Results are summarized in Table 1. Figure 2 shows the percentage time in clinically important hypoglycemia (<3.0 mmol/L) and percentage time in target (3.9–10 mmol/L) at baseline, at the 8-week endpoint and at the 16-week endpoint. In summary, the percentage time in hypoglycemia is significantly reduced from baseline to 8 weeks with RT-CGM and the improvement is maintained when continued for another 8 weeks, whereas in the flash group, the percentage time in hypoglycemia remains the same between baseline and the 8-week endpoint; however, when switched to RT-CGM, there is a significant reduction in hypoglycemia at the 16-week endpoint. There were no episodes of severe hypoglycemia requiring third-party assistance in either group throughout the study intervention periods.

**Table 1.** Change in Glucose Outcomes and Questionnaire Outcomes (Gold, HSF-II [Including HSF-II Behavior and Worry Subscores] and PAID Scores) from 8 Weeks to Endpoint at 16 Weeks with RT-CGM

*% Time in defined glucose range*	*RT-CGM group (*n* = 16)*			*Flash glucose monitoring group (*n* = 20)*			*Median change from 8 weeks to endpoint*		
	*At 8 weeks*	*Endpoint at 16 weeks*	P	*At 8 weeks*	*Endpoint at 16 weeks*	P	*RT-CGM group*	*FGM group*	P
<2.8 mmol/L	0.8 (0.2 to 1.7)	0.9 (0.3 to 1.5)	0.82	3.8 (3.0 to 6.4)	0.5 (0.2 to 1.4)	**<0.001**	0.0 (−0.9 to 0.3)	−3.1 (−4.6 to −2.4)	**<0.001**
<3.0 mmol/L	1.3 (0.4 to 2.8)	1.3 (0.8 to 2.5)	0.82	5.0 (3.7 to 8.6)	0.8 (0.4 to 1.9)	**<0.001**	0.02 (−1.2 to 0.5)	−4.0 (−4.8 to −2.9)	**<0.001**
<3.3 mmol/L	2.3 (0.9 to 4.4)	2.1 (1.4 to 4.4)	0.73	6.8 (4.8 to 11.7)	1.5 (0.7 to 2.8)	**<0.001**	0.4 (−1.7 to 0.9)	−4.8 (−6.2 to −3.2)	**<0.001**
<3.5 mmol/L	3.1 (1.5 to 5.9)	3.3 (2.2 to 6.7)	0.77	8.2 (6.0 to 13.2)	2.1 (1.2 to 4.0)	**<0.001**	0.4 (−2.3 to 1.7)	−5.3 (−7.6 to −3.3)	**<0.001**
<3.9 mmol/L	6.2 (3.1 to 8.7)	5.4 (3.9 to 9.7)	0.86	11.0 (8.2 to 17.0)	3.9 (2.4 to 6.7)	**<0.001**	0.4 (−0.2 to 2.1)	−6.6 (−9.4 to −3.7)	**<0.001**
>7.8 mmol/L	49.0 (43.9 to 56.8)	45.6 (40.4 to 63.5)	0.99	47.1 (37.4 to 53.5)	50.2 (44.7 to 57.8)	**0.007**	−0.3 (−5.5 to 6.6)	3.7 (−0.9 to 15.2)	**0.02**
>10 mmol/L	26.7 (17.5 to 36.1)	28.8 (15.8 to 46.3)	0.82	28.0 (18.0 to 32.1)	27.8 (23.0 to 34.3)	**0.02**	−1.0 (−5.4 to 8.5)	4.5 (−1.5 to 9.1)	0.18
>15 mmol/L	4.2 (1.2 to 8.8)	4.8 (1.4 to 9.1)	0.71	2.6 (1.2 to 5.1)	2.7 (2.3 to 5.0)	0.09	0.1 (−0.9 to 2.1)	0.6 (−0.3 to 2.8)	0.36
3.9 to 7.8 mmol/L	43.7 (38.1 to 47.8)	43.1 (32.1 to 54.0)	0.69	40.4 (34.7 to 45.3)	42.9 (34.9 to 49.8)	0.68	1.0 (−2.6 to 3.2)	2.2 (−5.2 to 4.7)	0.81
3.9 to 10 mmol/L	65.9 (54.1 to 74.8)	64.9 (49.2 to 73.9)	0.64	60.0 (54.5 to 67.8)	67.4 (56.3 to 72.4)	**0.02**	−1.0 (−4.4 to 4.1)	3.5 (−0.4 to 7.2)	**0.04**
Other outcomes									
Low blood glucose index	4.8 (2.8 to 6.3)	4.6 (3.8 to 5.9)	0.69	9.1 (7.2 to 10.7)	4.1 (3.0 to 4.9)	**<0.001**	0.2 (−1.4 to 1.2)	−4.8 (−6.2 to −2.3)	**<0.001**
Gold score	5.0 (3.0 to 5.0)	4.5 (3.0 to 6.0)	0.82	5.0 (3.5 to 6.0)	4.5 (3.0 to 5.5)	**0.04**	0 (−1 to 0.5)	0 (−1 to 0)	0.46
HbA1c mmol/mol	54.0 (46.0 to 62.0)	51.5 (47.0 to 58.0)	0.87	51.0 (48.5 to 59)	52.0 (49.5 to 60.5)	0.07	0.5 (−5.5 to 4.5)	2.0 (0.0 to 3.0)	0.49
HSF-II total score	54.0 (28.0 to 78.0)	47.0 (29.5 to 73.2)	0.07	42.0 (27.7 to 66.7)	38.0 (27.5 to 50.5)	0.15	−4.5 (−10.7 to 1.7)	−1.5 (−12.0 to 4.5)	0.94
HSF-II behavior	20.0 (11.0 to 28.5)	18.5 (10.2 to 24.7)	0.46	15.0 (11.2 to 27.7)	15.5 (11.2 to 22.7)	0.70	0.0 (−4.7 to 2.5)	−1.8 (−5.0 to 1.0)	0.41
HSF-II worry	32.0 (16.7 to 49.7)	29.5 (18.2 to 40.5)	0.18	31.0 (15.2 to 47.0)	21.5 (14.0 to 36.5)	**0.02**	−2.0 (−10.0 to 2.0)	−3.0 (−10.0 to 0.7)	0.51
PAID score	36.2 (10.0 to 52.5)	32.5 (12.5 to 38.7)	0.23	27.5 (15.6 to 55.0)	21.2 (17.8 to 38.4)	0.11	−1.2 (−8.7 to 2.5)	−1.2 (−7.1 to 0.9)	0.82

Results are expressed as median (IQR). *P* values of <0.05 are significant and highlighted in bold. Outcomes based on CGM data are derived from the last 28 days in each treatment period.

HFS-II, Hypoglycaemia Fear Survey; PAID, Problem Areas in Diabetes; RT-CGM, real-time continuous glucose monitoring.

The percentage time in target improves from baseline to the initial 8-week endpoint with both flash and RT-CGM, however, when the flash group is switched to RT-CGM there is further improvement in time in target at the 16-week endpoint, whereas in the RT-CGM the improvement is sustained, but no further significant improvement observed.

A significant improvement in HFS-II Worry subscore was seen when switching from flash to RT-CGM, whereas no further improvement was seen with continued use in the RT-CGM group. No significant difference in the reduction between groups was observed for HFS-II (including behavior and worry subscores) and PAID scores (Table 1).

## Discussion

Our pilot data suggest that switching from flash to RT-CGM has a significant beneficial impact on hypoglycemia outcomes and that continued use of RT-CGM maintains hypoglycemia risk/benefit in this high-risk population, across all predefined hypoglycemia thresholds. In addition, we have previously shown that percentage time in target improved with both flash and RT-CGM and here we demonstrate that switching from flash to RT-CGM achieves further benefit, whereas continuing with RT-CGM maintains benefit achieved over the first 8 weeks. Hypoglycemia fear resulting from worry improved in those who switched from flash to RT-CGM, however, the overall hypoglycemia fear reduction between groups was nonsignificant. The median Gold Score remained above 4 in both groups at the 16-week endpoint, suggesting that no clinically relevant improvement in hypoglycemia awareness was achieved by switching from flash to RT-CGM or continuing with RT-CGM for an extended time period.

This extension phase of the I HART CGM study is limited by small numbers and a short follow-up period. The glucose data at the 8-week endpoint in the flash and RT-CGM groups were derived from the Abbott Freestyle Libre device and from the Dexcom G5 RT-CGM device, respectively, but the final glucose data at the 16-week endpoint were derived from RT-CGM in both groups. This poses another limitation as previously highlighted^18^ when comparing outcomes based on two different technologies, where accuracy may not be equivalent and henceforth glucose outcomes may not be directly comparable. This applies when evaluating the difference from baseline to 8-week endpoint and from the 8-week to 16-week endpoint within the flash group. Both devices were used in accordance with the manufacturer's guidance. It has been reported that flash overestimates hypoglycemia,^19^ which may have contributed to the hypoglycemia results seen in our study, but not the time in range or hypoglycemia fear outcomes. Other data suggest that accuracy of flash and RT-CGM is comparable across a wide glucose range, including below hypoglycemia thresholds.^20,21^ We recognize the importance of a standard reference methodology when comparing different interstitial glucose monitoring technologies in clinical trials.

Our data support the NICE guidance^17^ that RT-CGM with alarms and alerts should be first line in people with T1D at high risk of hypoglycemia, but contradict the RMOC criteria for flash, which include people with T1D who have recently developed impaired awareness of hypoglycemia. There is an overlap between the two guidance documents, which may cause challenges in clinical practice. Although this is a pilot study with limitations, the data presented here continue to support the view that hypoglycemia risk, including frequency, severity, and awareness, must be assessed before offering diabetes technology interventions to people with T1D and that RT-CGM remains the appropriate first-line glucose monitoring option in hypoglycemia-prone individuals. The data presented here additionally suggest that there is a role for switching to RT-CGM in those already established on flash in this high-risk population and that the benefits of RT-CGM are sustained to 16 weeks.

## References

[1] Frier BM Hypoglycaemia in diabetes mellitus: epidemiology and clinical implications. Nat Rev Endocrinol 2014;10:711–722 25287289 10.1038/nrendo.2014.170

[2] Geddes J, Schopman JE, Zammitt NN, Frier BM Prevalence of impaired awareness of hypoglycaemia in adults with type 1 diabetes. Diabet Med 2008;25:501–504 18387080 10.1111/j.1464-5491.2008.02413.x

[3] Skrivarhaug T, Bangstad HJ, Stene LC, et al. Long-term mortality in a nationwide cohort of childhood-onset type 1 diabetic patients in Norway. Diabetologia 2006;49:298–305 16365724 10.1007/s00125-005-0082-6

[4] McCoy RG, Van Houten HK, Ziegenfuss JY, et al. Increased mortality of patients with diabetes reporting severe hypoglycemia. Diabetes Care 2012;35:1897–1901 22699297 10.2337/dc11-2054 PMC3425008

[5] Dahlquist G, Källén B Mortality in childhood-onset type 1 diabetes: a population-based study. Diabetes Care 2005;28:2384–2387 16186267 10.2337/diacare.28.10.2384

[6] Fidler C, Elmelund Christensen T, Gillard S Hypoglycemia: an overview of fear of hypoglycemia, quality-of-life, and impact on costs. J Med Econ 2011;14:646–655 21854191 10.3111/13696998.2011.610852

[7] Beck RW, Riddlesworth T, Ruedy K, et al. Effect of continuous glucose monitoring on glycemic control in adults with type 1 diabetes using insulin injections. JAMA 2017;317:371 28118453 10.1001/jama.2016.19975

[8] Lind M, Polonsky W, Hirsch IB, et al. Continuous glucose monitoring vs conventional therapy for glycemic control in adults with type 1 diabetes treated with multiple daily insulin injections the gold randomized clinical trial. JAMA 2017;317:379–387 28118454 10.1001/jama.2016.19976

[9] Tamborlane WV, Beck RW, Bode BW, et al. Continuous glucose monitoring and intensive treatment of type 1 diabetes. N Engl J Med 2008;359:1464–1476 18779236 10.1056/NEJMoa0805017

[10] Battelino T, Conget I, Olsen B, et al. The use and efficacy of continuous glucose monitoring in type 1 diabetes treated with insulin pump therapy: a randomised controlled trial. Diabetologia 2012;55:3155–3162 22965294 10.1007/s00125-012-2708-9 PMC3483098

[11] Battelino T, Phillip M, Bratina N, et al. Effect of continuous glucose monitoring on hypoglycemia in type 1 diabetes. Diabetes Care 2011;34:795–800 21335621 10.2337/dc10-1989 PMC3064030

[12] Heinemann L, Freckmann G, Ehrmann D, et al. Real-time continuous glucose monitoring in adults with type 1 diabetes and impaired hypoglycaemia awareness or severe hypoglycaemia treated with multiple daily insulin injections (HypoDE): a multicentre, randomised controlled trial. Lancet 2018;391:1367–1377 29459019 10.1016/S0140-6736(18)30297-6

[13] van Beers CAJ, DeVries JH, Kleijer SJ, et al. Continuous glucose monitoring for patients with type 1 diabetes and impaired awareness of hypoglycaemia (IN CONTROL): a randomised, open-label, crossover trial. Lancet Diabetes Endocrinol 2016;4:893–902 27641781 10.1016/S2213-8587(16)30193-0

[14] Vloemans AF, van Beers CAJ, de Wit M, et al. Keeping safe. Continuous glucose monitoring (CGM) in persons with type 1 diabetes and impaired awareness of hypoglycaemia: a qualitative study. Diabet Med 2017;34:1470–1476 28731509 10.1111/dme.13429

[15] Bolinder J, Antuna R, Geelhoed-Duijvestijn P, et al. Novel glucose-sensing technology and hypoglycaemia in type 1 diabetes: a multicentre, non-masked, randomised controlled trial. Lancet 2016;388:2254–2263 27634581 10.1016/S0140-6736(16)31535-5

[16] Regional T, Optimisation M, Libre F Regional Medicines Optimisation Committee (RMOC) Flash Glucose Monitoring Systems Position Statement. 2017 www.sps.nhs.uk/wp-content/uploads/2017/11/Flash-Glucose-monitoring-System-RMOC-Statement-final-2.pdf (accessed 91, 2018)

[17] National Institute for Health and Excellence (NICE). Type 1 diabetes in adults: diagnosis and management. 2015; pp. 1–80 www.nice.org.uk/guidance/ng17/resources/type-1-diabetes-in-adults-diagnosis-and-management-1837276469701 (accessed 91, 2018) 26334079

[18] Reddy M, Jugnee N, El Laboudi A, et al. A randomized controlled pilot study of continuous glucose monitoring and flash glucose monitoring in people with type 1 diabetes and impaired awareness of hypoglycaemia. Diabet Med 2018;35:483–490 29230878 10.1111/dme.13561 PMC5888121

[19] Kamecke U, Pleus S, Ulbrich S, et al. Time spent in the low glycemic range: differences between and within continuous glucose monitoring systems. Diabetes Technol Ther 2018;20:A54

[20] Freckmann G, Link M, Pleus S, et al. Measurement performance of two continuous tissue glucose monitoring systems intended for replacement of blood glucose monitoring. Diabetes Technol Ther 2018;20:541–549 30067410 10.1089/dia.2018.0105 PMC6080122

[21] Aberer F, Hajnsek M, Rumpler M, et al. Evaluation of subcutaneous glucose monitoring systems under routine environmental conditions in patients with type 1 diabetes. Diabetes Obes Metab 2017;19:1051–1055 28205324 10.1111/dom.12907

